# Regarding “osteoporosis screening and major osteoporotic fracture prediction by cranial computed tomography-derived hounsfield units: a multi-center study on opportunistic osteoporosis screening”

**DOI:** 10.1080/07853890.2025.2591239

**Published:** 2025-11-21

**Authors:** Renzhong Li, Kui Sun, Junchen Zhu

**Affiliations:** ^a^ Taizhou Hospital of Traditional Chinese Medicine, Taizhou, Jiangsu Province, China; ^b^ The Second Affiliated Hospital of Anhui University of Traditional Chinese Medicine, Hefei, Anhui Province, China; ^c^ Anhui Acupuncture Hospital, Hefei, Anhui Province, China

Dear Editor

The study by Wakolbinger-Habel et al. provides meaningful evidence that cranial CT-derived Hounsfield units (HU) can be used as an opportunistic marker for osteoporosis screening [1]. This multi-center work contributes valuable data on the relationship between cranial bone density and systemic bone mineral status, highlighting the potential of existing cranial CT scans to identify patients at risk of osteoporotic fractures. Nevertheless, several aspects deserve further discussion to strengthen the reproducibility and clinical interpretability of the results.

Because data were acquired from multiple Siemens CT platforms with heterogeneous reconstruction kernels and slice thicknesses, potential scanner-specific HU bias cannot be excluded. Even modest differences in reconstruction algorithms or dose modulation can lead to notable variability in measured HU, which may shift diagnostic thresholds. Future investigations could employ phantom-based or water-equivalent calibration and apply harmonization methods such as ComBat correction to minimize inter-scanner variability. Additionally, stratified analysis by scanner model or reconstruction parameters would clarify the robustness of the proposed 606 HU cutoff across different imaging systems [2].

The study’s threshold derivation relied exclusively on dual-energy X-ray absorptiometry (DXA) categories. While DXA remains the standard diagnostic reference, it does not fully capture fracture risk. Since the majority of the cohort had a history of major osteoporotic fracture, recalibrating HU thresholds based on fracture outcomes could improve their clinical relevance. Logistic or survival models using major osteoporotic fractures as endpoints would help determine whether HU predicts fractures independently of DXA, and whether integrating HU into established risk models such as FRAX adds incremental value for clinical decision-making [3].

From an integrative medicine perspective, incorporating functional assessments may further refine cranial HU-based fracture risk prediction. Traditional Chinese Medicine (TCM) emphasizes the interconnection between muscle strength, postural stability, and bone health in its orthopedic and rehabilitation Practice [4]. Evidence demonstrates that TCM-based multimodal interventions—including Tai Chi exercise, acupuncture, and herbal therapies—can improve bone mineral density, enhance muscle endurance, and reduce fracture risk in osteoporotic patients [5]. Future studies tracking HU changes following such interventions could help establish HU as a dynamic biomarker reflecting both structural and functional bone quality.

In summary, this study establishes an important foundation for opportunistic osteoporosis detection using cranial CT. Building on this work, future research should focus on scanner harmonization, outcome-based validation, and the integration of functional and complementary-medicine perspectives. Such refinements would help transition cranial HU from a purely diagnostic parameter to a practical indicator guiding personalized bone health management and early fracture prevention.

## Data Availability

Data sharing is not applicable to this article as no data were created or analysed in this study.
